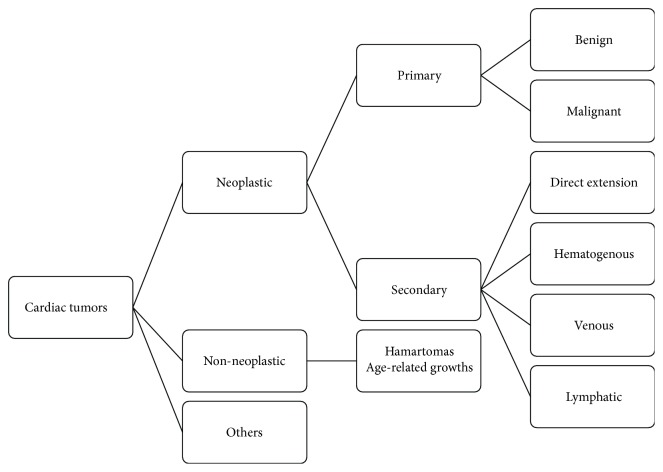# A Rare Case of an Ectopic Liver Presenting as Right Atrial Mass

**DOI:** 10.1155/2019/4103827

**Published:** 2019-05-15

**Authors:** Adhirath Doshi, Dhaval Shah, Shradha Gupta, Deepika Panday, Arie Farji-Cisneros, Eddison Ramsaran, Robert Bojar

**Affiliations:** ^1^Division of Cardiology, St. Vincent Hospital, Worcester, MA, USA; ^2^Department of Internal Medicine, St. Vincent Hospital, Worcester, MA, USA; ^3^Department of Anesthesia, St. Vincent Hospital, Worcester, MA, USA; ^4^Department of Cardiology, Reliant Medical Group, Worcester, MA, USA; ^5^Division of Cardiothoracic Surgery, St. Vincent Hospital, Worcester, MA, USA

## Abstract

Ectopic liver tissue is commonly observed in the abdominal cavity in adjacent organs. Extension of hepatic tissue into the intrathoracic cavity is rarely reported. We present the case of a 46-year-old woman with a 2.1 × 1.8 cm mass confirmed by transesophageal echocardiogram to be at the right atrial and inferior vena cava junction that was initially thought to be a myxoma which prompted surgical excision but subsequently identified as ectopic liver by histology.

## 1. Introduction

Primary cardiac tumors are rare with an autopsy frequency of 0.001-0.03% [1], whereas the incidence of secondary cardiac tumors is nearly 20 times higher [1–4]. The pathology of an intracardiac mass cannot always be determined by echocardiography, nearly always mandating surgical removal to prevent embolization and confirm the diagnosis. We herein report a rare case in literature of ectopic liver tissue presenting as two separate masses in the right atrium (RA) and inferior cava (IVC) [5–7].

## 2. Case

**Figure 1:**
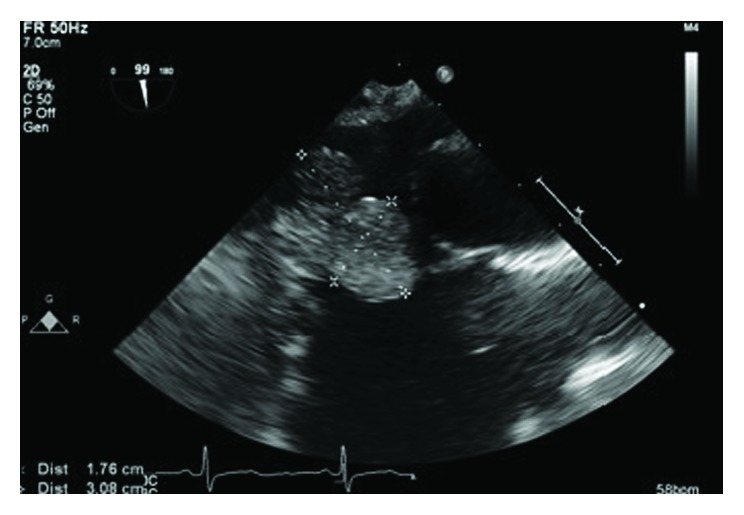
TEE showing the bilobed mass measuring 2.1×1.8 cm in the right atrium.

**Figure 2:**
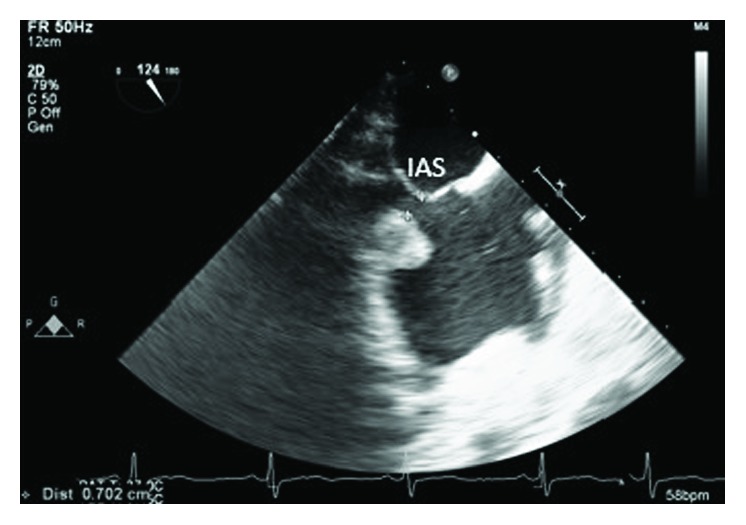
TEE showing the mass is not attached to the interatrial septum (IAS).

**Figure 3:**
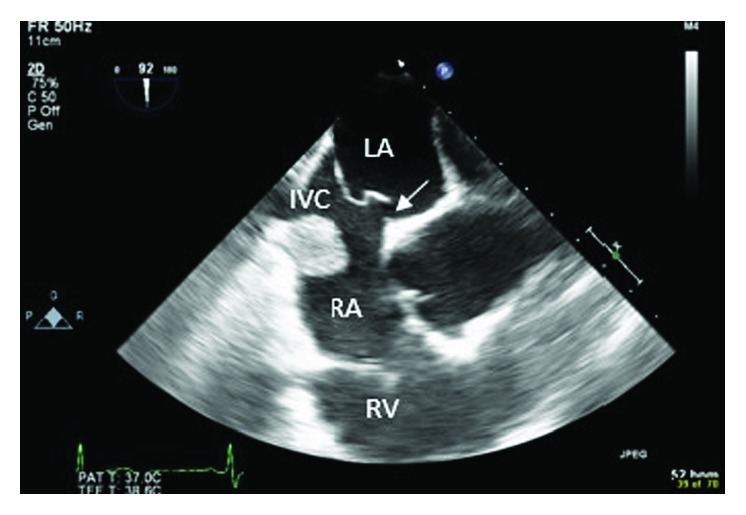
TEE showing extension of the mass into the IVC and a secundum ASD (white arrow). Left atrium: LA; right atrium: RA; right ventricle: RV.

A 46-year-old woman with morbid obesity, hypertension, hyperlipidemia, active smoking, and bipolar disorder was evaluated for paroxysmal atrial fibrillation as an outpatient. A transthoracic echocardiogram showed a right atrial mass close to the RA-IVC junction. A transesophageal echocardiogram confirmed the presence of a pedunculated right atrial mobile bilobular mass measuring 2.1 × 1.8 cm, not arising from the interatrial septum with extension into the inferior vena cava (Figures 1–3). A secundum atrial septal defect was also identified. The patient was referred to cardiac surgery for resection of a presumed right atrial myxoma given the possibility of embolization. The intraoperative TEE suggested the presence of one bilobulated mass arising low in the right atrium.

**Figure 4:**
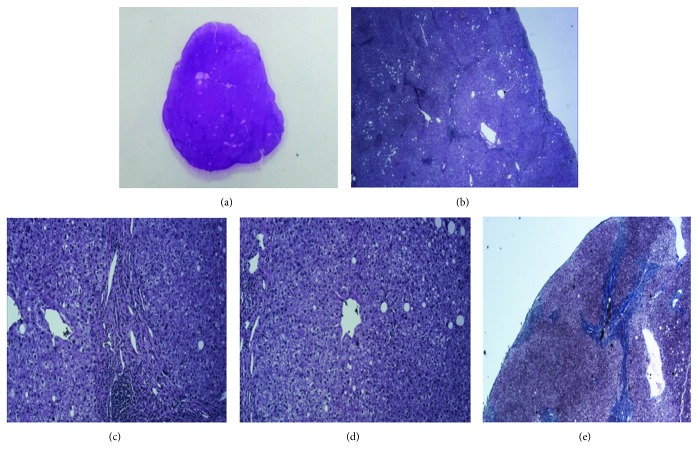
Histology of ectopic liver in the heart. (a) Photomicrograph with hematoxylin and eosin stain showing a 1.6 cm mass. (b) Normal liver parenchyma at 2x magnification. (c) Portal tract at 10x magnification with lymphocytic infiltration of parenchyma. (d) Central vein at 10x magnification. (e) Normal liver parenchymal architecture highlighted by trichrome stain at 2x magnification.

Using aortic and bicaval cannulation, a right atriotomy was performed and a discrete 2 × 3 cm mass was removed from the IVC-RA junction near the Eustachian valve. The right atrium was closed and the patient weaned from bypass. The specimen did not appear to be a myxoma, so it was sent for frozen section and interpreted as being ectopic liver tissue. In the interim, a repeat TEE showed an additional discrete 2 × 2 cm mass in the IVC near the hepatic veins. Femoral venous cannulation was then performed to allow for visualization of the IVC below the pericardial reflection. Through the same right atriotomy and using additional suction directly in the IVC, the IVC mass was visualized and resected. Both specimens were interpreted as showing partially encapsulated liver parenchyma with mild steatosis, fibrosis, ductal proliferation, and periductal chronic inflammation, consistent with ectopic hepatic tissue (Figure 4).

Despite a brief period of cardiopulmonary bypass, the patient had persistent hypoxemic respiratory failure ascribed to her morbid obesity and underlying lung disease. She also developed extensive left lower extremity deep venous thrombosis from a presumed heparin-induced thrombocytopenia for which she was treated with argatroban and placement of an IVC filter. Because of chronic respiratory failure, she underwent tracheostomy and feeding tube placement, and she was placed on warfarin for intermittent atrial fibrillation due to her CHA_2_DS_2_-VASc score of 4. She gradually improved and was transferred to rehab where her tracheostomy tube and PEG tube were removed. A few months later, she looked quite well during an office visit.

## 3. Discussion

The differential diagnosis of intracardiac masses include benign tumors, primary or metastatic malignant tumors, and thrombus (Figure 5) [8]. The location, attachment site, size, shape, and underlying patient characteristics are helpful in prioritizing the diagnosis. Myxomas are the most common benign tumor located in the right atrium, and renal cell carcinoma is the most common tumor located at the RA-IVC junction [9]. Symptoms from the right atrial masses may result from obstruction of blood flow through the tricuspid valve mimicking tricuspid stenosis, distal embolization to the lungs, or in the case of an atrial septal defect, embolization to the systemic circulation.

The incidence of ectopic liver described by laparoscopic and autopsy studies is less than 0.5% [10–12]. In the absence of trauma, most cases of ectopic liver are believed to be congenital in origin. The liver develops from the ventral outgrowth of the foregut during the 4th week of gestation. The exact pathogenesis of ectopic liver is unclear and Trocciola et al. [5] offer the following hypotheses: (1) development from the second liver bud, (2) development from the original liver bud with migration of cells to form an accessory liver that is connected to the original liver by a stalk, (3) incomplete atrophy or regression of the developing liver lobes, and (4) migration of nests of hepatic cells outside the developing liver. Ectopic liver is usually found in an intraabdominal location but rarely in the intrathoracic cavity or heart. Ectopic liver is found to have increased neoplastic potential [13] and should be resected when identified, although rarely is the diagnosis suspected beforehand.

## 4. Conclusion

Isolated right atrial masses are rare and most are discovered incidentally on echocardiography or CT scanning. Imaging studies are useful to identify their size, mobility, and sites of attachment. Resection should be guided by symptoms and/or embolic potential of these masses. The location, attachment, texture, and consistency can provide a clue to the origin of these masses. Intracardiac ectopic liver is an extremely rare diagnosis, but an important one to make primarily due to an increased incidence of carcinogenesis in the ectopic tissue. In our patient, the presence of a separate tumor mass in the high inferior vena may have been a clue that this mass was not a myxoma or a renal cell carcinoma. Resection of caval tumors can be a technical challenge [14], depending on the extent of the tumor. In our patient, it was not immediately recognized that there were two masses, so the initial cannulation technique was inadequate to resect the IVC mass. Use of femoral venous cannulation was therefore required for better visualization.

## Figures and Tables

**Figure 5:**